# Examining the Evidence on the Statistics Prerequisite for Admission to Doctor of Nursing Practice Programs: Retrospective Cohort Study

**DOI:** 10.2196/57187

**Published:** 2024-09-09

**Authors:** Ha Do Byon, Sunbok Park, Beth A Quatrara, Jessica Taggart, Lindsay Buford Wheeler

**Affiliations:** 1 School of Nursing University of Virginia Charlottesville, VA United States; 2 Center for Teaching Excellence University of Virginia Charlottesville, VA United States

**Keywords:** Doctor of Nursing Practice, admission prerequisite, statistics requirement, biostatistics, nursing education

## Abstract

**Background:**

Doctor of Nursing Practice (DNP) programs in the United States confer the highest practice degree in nursing. The proportion of racial and ethnic minority DNP students, including those of Asian descent, keeps increasing in the United States. Statistics is commonly required for DNP programs. However, there is insufficient evidence regarding the number of years within which statistics should be taken and the minimum grade required for admission to the program.

**Objective:**

This study aimed to examine the associations of statistics prerequisite durations and grades for admission with the course performances within the DNP program. We also explored whether a postadmission statistics overview course can prepare students for a DNP statistics course as well as a required statistics prerequisite course.

**Methods:**

A retrospective cohort study was conducted with a sample of 31 DNP students at a large university in the Mid-Atlantic region. Statistical analysis of data collected over 5 years, between 2018 and 2022, was performed to examine the associations, using Spearman rank correlation analysis and Mann-Whitney *U* test (*U*).

**Results:**

The performance of students in a DNP statistics course was not associated with prerequisite duration. There was no significant association between the duration and the DNP statistics course letter grades (ρ=0.12; *P*=.66), neither with exam 1 (ρ=0.03; *P*=.91) nor with exam 2 scores (ρ=0.01; *P*=.97). Prerequisite grades were positively associated with exam 1 grades (ρ=0.59; *P*=.02), but not exam 2 (ρ=0.35; *P*=.19) or course grades (ρ=0.40; *P*=.12). In addition, no difference was found in the performance of students whether meeting the prerequisite requirements or taking a 1-month, self-paced overview course (exam 1: *U*=159, *P*=.13; exam 2: *U*=102, *P*=.50; course letter grade: *U*=117, *P*=.92).

**Conclusions:**

No evidence was found to support the need for limits on when prerequisites are completed or grade requirements. Opting for a statistics overview course after admission can serve as a viable alternative to the statistics prerequisite, effectively preparing students for advanced quantitative data analysis in a DNP program.

## Introduction

Doctor of Nursing Practice (DNP) programs in the United States prepare nurse leaders, conferring the highest practice degree in nursing. The proportion of racial and ethnic minority DNP students, including those of Asian descent, increased from 21% in 2010 to 37% in 2020 [1]. Several countries in East and Southeast Asia have begun implementing practice-oriented nursing doctorate programs, similar to DNP programs in the United States [2]. The American Association of Colleges of Nursing (AACN) documents that guide the development of DNP program curricula emphasize the importance of DNP graduates being prepared to lead evidence-based practice [3]. To fulfill this, DNP graduates should possess the competence to translate research into practice, evaluate evidence, apply research in decision-making, and implement viable clinical innovations to effect practice change [4].

Integration of research into clinical practice for evidence-based practice requires a firm understanding of statistics. Insufficient knowledge and reasoning skills in statistics can lead to inaccurate interpretation and application of knowledge [4]. Accordingly, it is common that DNP programs require statistics as an admission prerequisite. A total of 17% of DNP programs in the United States mandate applicants to complete a graduate-level statistics course before admission [5]. Some programs include statistics as a core course in their DNP curriculum [6], often requiring undergraduate-level statistics as a prerequisite.

While completion of a statistics course before admission to DNP programs is frequently required, there has been little evidence or consensus about the specific timeframe within which this prerequisite should be fulfilled [5] or whether prerequisites are even needed [7]. Candidates are largely affected by the existence and duration of this requirement; a tight, short timeframe may discourage them from applying to the program as it may mean they have to retake a statistics course to meet the requirement [8]. Such a duration requirement, when unnecessary, is disadvantageous to both applicants seeking to advance their education and to schools aiming to expand the qualified applicant pools [8]. Previous research has indicated limited evidence linking prerequisite coursework to subsequent performance [9,10]. There is no published evidence showing a differential effect of prerequisite statistics on the admission or academic performance of racial and ethnic minority DNP students. Furthermore, admission requirements have changed over time, implying that specific year criteria may not always be a good standard [7]. As the specific duration criteria of prerequisites vary across the DNP programs and limited evidence supports the need for prerequisite requirements, it is vital to address the existing gaps in prerequisite courses and to examine the evidence supporting the criteria [11].

Therefore, this study aimed to investigate the questions: (1) Is the time elapsed since students’ completion of the statistics prerequisite (duration) associated with their performance in a DNP statistics course? (2) Are the grades of the statistics prerequisite course associated with students’ performance in a DNP statistics course? and (3) Does a postadmission statistics overview course prepare students for a DNP statistics course as well as a required statistics prerequisite course?

## Methods

### Study Design and Sample

We conducted a retrospective cohort study that involved the analysis of data collected over 5 years, between 2018 and 2022. The unit of analysis was data from individuals. The data sources consisted of the transcripts and class artifacts of the students who were enrolled in a DNP program–required statistics course at a large university in the Mid-Atlantic region. We did not collect their demographics for anonymity. The DNP program in this study is designed to empower nurses to practice at the highest level of clinical practice and improve systems and safety as nurse leaders. The executive, hybrid format allows students to attend in-person classes once a month, with the balance of online learning. At the end of the program, students complete a quality improvement scholarly project that synthesizes and demonstrates the knowledge and skills they have learned throughout the program. Statistics play a key role in analyzing data for this scholarly project. While the DNP program required completing a prerequisite statistics course with a grade of at least B within 5 years of the program start, it also allowed the students who had not met either the grade requirement or the duration requirement of 5 years to alternatively take a summer statistics overview course. The 1-month, online, asynchronous, self-paced summer overview course was supervised by a faculty member and contained 5 modules (research design, sampling, measurement, descriptive statistics, and inferential statistics). In order to pass the course, the students were required to demonstrate at least 80% proficiency with the content through an end-of-the-course exam that they could take a maximum of 2 times. DNP statistics course grades were accessed by the course professor (HDB). The grades and the years and semesters of the prerequisite statistics course were obtained through the School of Nursing Office of Admissions and Student Services. The passing status of the summer overview course was obtained from the Director of the DNP program.

### Measures

#### Duration Since Statistics Prerequisite

Students started taking the DNP statistics course at various elapsed times after completion of the prerequisite statistics course. To compute the duration of this elapsed time (DET), first, the years and months were identified for (1) when students had completed the prerequisite statistics courses based on their transcript (May for the spring semester, August for the summer semester, and December for the fall semester) and (2) when students had started taking the DNP statistics course (August). Then, we calculated the DET in months by subtraction between the 2 time points.

#### Doctor of Nursing Practice Statistics Course Performances

In total, 3 DNP statistics course artifacts were used to measure the course performance of students: exam 1 and exam 2 scores (both with a possible score range of 0 to 15), and course letter grades (1=B–, 2=B, 3=B+, 4=A–, 5=A, and 6=A+). No study participants received a grade below B in the course.

The contents of the 2 exams were not cumulative. Exam 1 occurred at the midterm and exam 2 at the end of the semester. Each included 25 multiple-choice questions. The exams assessed students’ understanding of the statistical theories and interpretation of the results from various statistical tests (eg, descriptive statistics, *t* tests, one-way ANOVA, repeated measures ANOVA, analysis of covariance, multiple linear regression, logistic regression, and nonparametric tests). The course letter grades also reflected other course artifacts (eg, a statistical analysis critique paper) that were excluded from this study due to a lack of immediate relevance to the prerequisite statistics grades. The course was taught by the same faculty member during the entire 5-year study period. The faculty maintained the other format (eg, exam length) and the difficulty level of the exams across cohorts of students, supported by comparable exam scores and course grades, on average, over the years.

#### Prerequisite Statistics Performance

The letter grades of the statistics courses in the transcripts that students had submitted for admission were used to measure the performance of the students for the prerequisite statistics course (1=B–, 2=B, 3=B+, 4=A–, 5=A, and 6=A+). The DNP program required a grade of at least B– for admission.

### Ethical Considerations

All participants were informed of the purpose, content, process, and potential risks and benefits of the study. The informed consents were voluntarily obtained, and participants were informed that they could withdraw from the study whenever they wanted. No compensation was provided for study inclusion. The data used in this study were stored in the institutional encrypted storage. All study data were accessible only to the approved researchers. This study was approved by the University of Virginia Institutional Review Board (#5525) and adhered to ethical standards.

### Statistical Analysis

Descriptive and inferential statistics were calculated using IBM SPSS Statistics (version 28) [12]. Frequencies and proportions in percent for categorical variables and their means (SDs) and medians (IQRs) for continuous variables were computed. A Spearman rank correlation analysis was used to explore the association between the DET and their DNP statistics course performance (study question 1) and statistics prerequisite course grades and students’ DNP statistics course performance (study question 2) because the study data did not meet the normality assumption. A Mann-Whitney *U* test was used to investigate differences in DNP statistics course performance based on the completion of a statistics overview course or required statistics prerequisite (study question 3). The significance level for all statistical tests was set at the conventional level of *P*=.05.

## Results

### Sample Characteristics

Out of 86 students who had taken the DNP program statistics course and were invited to the study, a total of 31 (36%) students agreed to participate. From the 31 student participants in the sample, 16 (52%) students met the 5-year statistics prerequisite requirement for admission, and 15 (48%) students successfully completed the summer overview course. There were no missing data.

Among the 16 students who fulfilled the 5-year statistics requirement, the mean DET between taking the prerequisite statistics course and the DNP statistics course was 39.6 (SD 19.7; range 12-80) months, or approximately 3.5 years. Most of them (11/16, 69%) received an A in their prerequisite statistics course. In the total sample of 31 students, which was used to answer study question 3, the distribution of the letter grades in the DNP statistics course was left skewed, with predominantly A+ or A. Their mean scores for exam 1 and exam 2 were 13.4 (SD 1.5) out of 15 and 13.1 (SD 1.5) out of 15, respectively (Table 1).

**Table 1:** Sample characteristics of the study participants.

Characteristics			Descriptive statistics
**Transcript grade of the prerequisite statistics course (n=16), n (%)**			
	A		11 (69)
	B		4 (25)
	B–		1 (6)
**Duration (months elapsed from prerequisite; n=16)**			
	Mean (SD)		39.6 (19.7)
	Median (IQR)		34 (27.0-53.5)
**Year taking the DNP^a^ statistics course (n=31), n (%)**			
	2018-2019		5 (16)
	2020-2021		19 (61)
	2022		7 (23)
**DNP statistics course grade (n=31),** **n (%)**			
	A+		15 (48)
	A		10 (32)
	A–		4 (13)
	B+		2 (6)
**DNP statistics course performances (maximum score=15; n=31)**			
	**Exam 1**		
		Mean (SD)	13.4 (1.5)
		Median (IQR)	13.8 (12.8-14.5)
	**Exam 2**		
		Mean (SD)	13.1 (1.5)
		Median (IQR)	13.7 (12.0-14.4)

^a^DNP: Doctor of Nursing Practice.

### Association Between Duration and Class Performance

There was no significant association between the DET and the DNP statistics course letter grades (ρ=–0.12; *P*=.66). Also, the DET was neither significantly associated with exam 1 (ρ=0.03; *P*=.91) nor exam 2 scores (ρ=–0.01; *P*=.97; Table 2). There was no difference between those who had a DET within 5 years and more than 5 years in their scores of exam 1 (14.2 out of 15 vs 14.2 out of 15), exam 2 (13.2 out of 15 vs 13.6 out of 15), and course grades (12/13, 92% students earned A– or above vs 3/3, 100% earned A– or above; Table 3).

**Table 2:** Association of class performance with prerequisite duration and transcript grade (n=16).

Class performance			Duration		Transcript grade
**Exam 1 score**					
	ρ	0.03		0.59	
	*P* value	.91		.02	
**Exam 2 score**					
	ρ	–0.01		0.35	
	*P* value	.97		.19	
**DNP^a^ course letter grade**					
	ρ	–0.12		0.40	
	*P* value	.66		.12	

^a^DNP: Doctor of Nursing Practice.

**Table 3:** Comparison of the course performances by prerequisite duration (≤5 years vs >5 years; n=16).

Course performance		Duration	
		≤5 years (n=13)	>5 years (n=3)
**Exam 1**			
	Mean (SD)	14.2 (0.9)	14.2 (0.9)
	Median (IQR)	14.4 (13.8-15.0)	14.4 (13.8-14.7)
**Exam 2**			
	Mean (SD)	13.2 (1.4)	13.6 (1.9)
	Median (IQR)	13.8 (12.6-13.8)	14.4 (12.9-14.7)
**Letter grade, n (%)**			
	A+	6 (46)	2 (67)
	A	4 (31)	0 (0)
	A–	2 (15)	1 (33)
	B+	1 (8)	0 (0)

### Association Between Prerequisite Performance and Class Performance

There was a significant and large association between letter grades in the prerequisite statistics course and exam 1 scores in the DNP statistics course (ρ=0.59; *P*=.02). However, the prerequisite letter grades were not significantly associated with exam 2 scores and the DNP statistics course letter grades, respectively (ρ=0.35, *P*=.19; ρ=0.40, *P*=.12). All other effect sizes were close to 0 (Table 2).

### Comparison Between Statistics Prerequisite and Overview Course

For the 31 students in the full sample, there were no differences in all 3 DNP statistics course performance measures between those who met the statistics prerequisite requirement and those who took the statistics overview course (exam 1: *U*=159, *P*=.13; exam 2: *U*=102, *P*=.50; course letter grade: *U*=117, *P*=.92). Exam 1 scores were slightly higher (14.2 out of 15 vs 13.5 out of 15) among those who met the prerequisite, but the difference was not significant (Table 4).

**Table 4:** Comparison between those who met the statistics prerequisite and those who took the overview course (n=31).

			Exam 1		Exam 2		Course letter grade
**Total (n=31)**							
	Mean (SD)	13.9 (1.1)		13.3 (1.7)		5.2 (0.9)	
	Median (IQR)	14.4 (13.2-14.7)		13.9 (12.6-14.4)		5.0 (5.0-6.0)	
**Prerequisite group (n=16)**							
	Mean (SD)	14.2 (0.9)		13.3 (1.4)		5.2 (1.0)	
	Median (IQR)	14.4 (13.5-15.0)		13.8 (12.0-14.1)		5.5 (4.5-6.0)	
**Overview course group (n=15)**							
	Mean (SD)	13.5 (1.2)		13.3 (2.0)		5.3 (0.9)	
	Median (IQR)	13.8 (12.9-14.4)		14.4 (12.6-14.7)		5.0 (5.0-6.0)	
*U* ^a^			159.0		102.0		117.0
*P* value			.13		.50		.92
Pearson correlation coefficient (*r*) (effect size)			0.28		0.13		0.02

^a^Mann-Whitney *U* test

## Discussion

We did not find a significant association between the duration after students’ completion of the statistics prerequisite and their DNP statistics course performance. When a DNP program requires statistics as an admission prerequisite, a duration requirement is often attached (eg, 5 years) [11]. The rationale is that the knowledge of statistics has a limited shelf-life. However, there is no known evidence after what year the knowledge degrades significantly enough to warrant retaking a course. In our study, there was no such critical time point. Furthermore, our study result showed no overall association of duration with performance in the higher-level statistics course. Although the duration in our study sample only spanned from 12 to 80 months, there is little reason to suppose a significant association beyond the period.

Likewise, we did not find a correlation between course grades in the prerequisite statics course and performance in a DNP statistics course. Although exam 1 scores were positively associated with the prerequisite grades, the association did not continue for exam 2 or the overall course grades. In this context, the disadvantage for the students with lower prerequisite course grades was overcome by the end of the semester. One possible explanation for this is that the attenuation of the significant association over the semester might be related to what and how students and faculty contribute to the learning process. The faculty might have put more effort into helping the students with lower prerequisite grades, and at the same time, those students might have sought more academic support from the faculty. However, future research is needed to verify these claims. It also needs to be reiterated that all participating students in this study had at least a B– grade in their prerequisite statistics course, as this was an admission requirement. Therefore, the absence of the correlation over time may not apply to candidates who earned lower than B– in their prerequisite course. Nevertheless, our study findings highlight the necessity for evidence-based criteria regarding statistics requirements, which may differ from current DNP admission practices in many programs.

It is noteworthy that we did not find a difference in students’ performance whether they met the prerequisite requirements or took an alternative overview course. A faculty-supervised, 1-month, self-paced, online statistics overview course was as effective in preparing the students for the DNP statistics course as a statistics course taken as a prerequisite. The effectiveness of the statistics overview course presents a novel avenue diverging from the conventional statistics prerequisite in DNP programs. This pathway allows these programs to gauge the readiness of students for their enrollment in an advanced graduate-level statistics course within the program itself, eliminating the need for them to undertake an additional statistics course before joining the program. Also, this strategic initiative has the potential to foster greater equity and inclusivity within DNP program learning cohorts. By affording students the chance to familiarize themselves with essential content over a condensed timeframe, the approach eliminates the necessity of seeking an external course. Providing such an overview course after students are admitted, rather than requiring them to take an external course as a prerequisite to admission, can align with and strengthen increasing diversity, equity, and inclusion efforts at academic institutions. Our study had several limitations. The study sample size was small and recruited from one DNP program. We also did not collect data on potential confounding variables such as student sociodemographics (age, social support, work status, etc) and their academic tracks (part-time and full-time). Further research is needed to explore whether and how these student characteristics influence the outcomes. Also, collaborative or comparative studies with other health care education programs can be conducted to gain insights from diverse perspectives.

In conclusion, we did not find evidence to support requiring candidates to complete a statistics prerequisite course for admission within a fixed number of years. Instead, there is a need to establish evidence-based criteria regarding statistics requirements. Alternatively, offering a statistics overview course can be an effective way to prepare students for a higher-level statistics course in DNP programs. With support, students have the potential to achieve desirable learning outcomes.

## Abbreviations

AACN: American Association of Colleges of Nursing
DET: duration of elapsed time
DNP: Doctor of Nursing Practice

## Data Availability

The datasets generated during and/or analyzed during this study are not publicly available due to confidentiality.
